# Lumiflavin Reduces Cisplatin Resistance in Cancer Stem-Like Cells of OVCAR-3 Cell Line by Inducing Differentiation

**DOI:** 10.3389/fonc.2022.859275

**Published:** 2022-05-20

**Authors:** Ruhui Yang, Bingjin Liu, Mingyue Yang, Feng Xu, Songquan Wu, Shufang Zhao

**Affiliations:** ^1^School of Medicine and Pharmaceutical Engineering, Taizhou Vocational and Technical College, Taizhou, China; ^2^Department of Pharmacology, Lishui University School of Medicine, Lishui, China; ^3^Clinical Department, China Medical University, Shenyang, China; ^4^Department of Immunology, Lishui University School of Medicine, Lishui, China; ^5^Molecular Biology Laboratory, Lishui University School of Medicine, Lishui, China

**Keywords:** lumiflavin, ovarian cancer, cancer stem-like cells, drug resistance, phenotypic differentiation

## Abstract

Ovarian cancer stem-like cells (CSCs) play a vital role in drug resistance and recurrence of ovarian cancer. Inducing phenotypic differentiation is an important strategy to enhance the effects of chemotherapy and reduce the drug resistance of CSCs. This study found that lumiflavin, a riboflavin decomposition product, reduced the development of CSC resistance and enhanced the chemotherapy effect of cisplatin (DDP) on CSCs in DDP-resistant ovarian cancer OVCAR-3 cell line (CSCs/DDP) and was related to the induction of CSC phenotypic differentiation. Results showed that the development of DDP-resistant OVCAR-3 cells was related to the increase in the proportion of CSCs/DDP, and the treatment with lumiflavin reduced the DDP-resistance levels of OVCAR-3 cells and proportion of CSCs/DDP. Further investigation found that lumiflavin synergistic with DDP increased apoptosis, decreased mitochondrial membrane potential, and inhibited the clonal formation of CSCs/DDP. Meanwhile, *in vivo* experiments showed that lumiflavin dose-dependently enhanced the chemotherapy effect of DDP on tumor-bearing nude mice inoculated by CSCs/DDP. Lumiflavin treatment also reduced the ratio of CD133+/CD177+ to CD44+/CD24 cells, which is the identification of CSCs, in CSCs/DDP. In addition, transcriptome sequencing results suggested that the role of lumiflavin was related to the notch and stem cell pathway, and Western blot analysis showed that lumiflavin inhibited the protein expression of notch signaling pathway in CSCs/DDP. In conclusion, lumiflavin reduces the development of the drug resistance of OVCAR-3 cell and increases the sensitivity of CSCs/DDP to DDP by inducing phenotypic differentiation, which may have a potential role in the chemotherapy treatment of ovarian cancer.

## Introduction

Although most patients with advanced ovarian cancer respond well to paclitaxel and platinum to the initial chemotherapy, 70% – 85% of patients, including those who have a significant effect on the treatment, still relapse within a few years after receiving systemic chemotherapy or cytoreductive surgery (1). Ovarian cancer stem-like cells (CSCs), a subgroup of tumor cells that exhibit the ability of stem cells (i.e., self-renewal, multidirectional differentiation, and the ability to cause tumorigenesis and metastasis) in tumor tissues, are considered responsible for the generation and recurrence of drug resistance in ovarian cancer cells (2, 3).

Currently, the failure to kill CSCs effectively is an important reason for ovarian cancer relapse after chemotherapy (4). CSCs are generally in the G0 cell phase, with high telomerase activity and DNA repair ability, as well as anti-apoptotic genes to evade chemotherapy damage; CSCs that have not been killed by chemotherapy in ovarian cancer become the “seeds” of recurrence and metastasis (5–8). The CSC hypothesis explains the mechanism of tumor recurrence and drug resistance. Therefore, therapies that target CSCs selectively may offer a greater promise for treating ovarian cancer.

**Figure 1:**
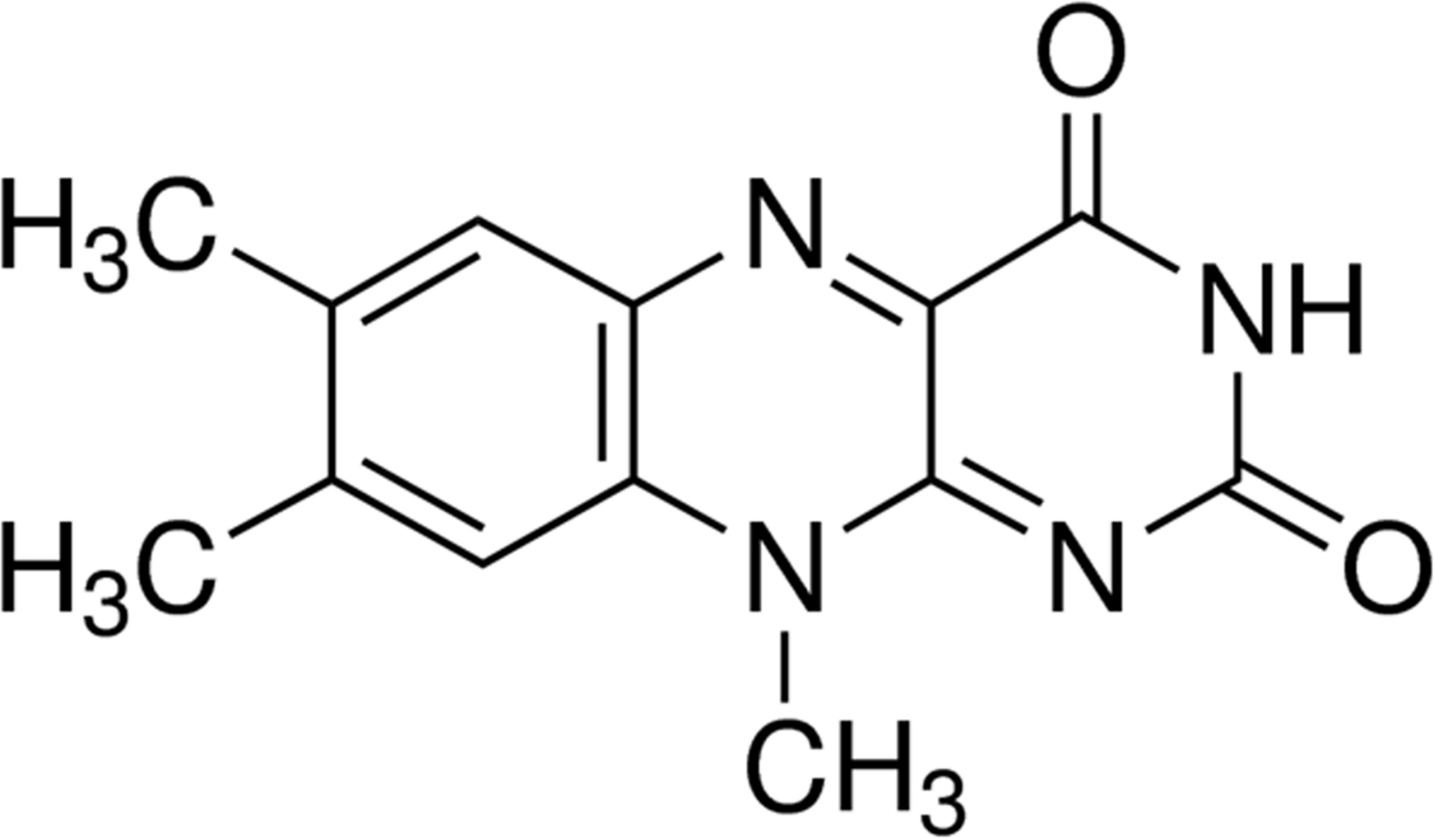
Chemical structure of lumiflavin.

Previous studies in our laboratory have shown that riboflavin is an important factor in maintaining the characteristics of CSCs (9, 10). Lumiflavin, a flavin analogue (**Figure 1**), is a competitive inhibitor of riboflavin. Our previous studies have proved that lumiflavin reduced the enrichment of ovarian cancer CSCs to riboflavin, lessened the tolerance of CSCs to DDP, improved the damage effect of chemotherapy drugs, and enhanced the radiotherapy effect on CSCs (11). However, the effect of lumiflavin on the development of drug resistance in ovarian cancer, especially on drug-resistant CSCs, is still vague. In this study, the CSCs of OVCAR-3 cell line was used to verify the effect of lumiflavin on the formation of drug resistance in ovarian cancer.

Inducing differentiation is an important strategy to kill CSCs effectively and reduce the formation of drug resistance (12, 13), such as morphogen-driven signaling cascade (14), and epigenetic differentiation therapy (15). Clinically common differentiation-inducing agents, such as all-trans retinoic acid (ATRA), a metabolic intermediate of vitamin A, reduce the invasiveness and tumorigenicity of CSCs by blocking angiogenic cytokines in glioblastoma (16). It can also induce the differentiation of breast cancer CSCs, reduce the invasion and migration of cells, and improve the sensitivity to tumor treatment (17). Previous studies showed that the effect of lumiflavin was related to its reduction in the CSC proportion (11, 18). Therefore, we speculated that lumiflavin may affect the differentiation of ovarian cancer CSCs. To assess this hypothesis, the present study investigated the effects of lumiflavin on DDP resistance of ovarian cancer CSCs and the relationship between these effects and phenotypic differentiation of CSCs. Results suggest that lumiflavin has therapeutic value in alleviating the development of drug resistance in ovarian cancer.

## Materials and Methods

### Cell Lines, Drugs, Animals, Reagents, and Equipment

Human ovarian cancer cell lines OVCAR-3 (Shanghai Cell Bank of Chinese Academy of Sciences), Lumiflavin (98%, TRC, Canada), DDP (National Institutes for Food and Drug Control of China), Accuri C6 flow cytometry (BD Biosciences, U.S.A) and Western blot system (Bio-Rad, USA), Specific pathogen-free BALB/c nude female mice with an age of approximately 6 weeks and weight of 18–22 g were provided by Shanghai Slack Laboratory Animal Co., Ltd. (Shanghai, China). The mice were housed in a temperature-controlled room under a 12 h dark/light cycle and were allowed access to food and water ad libitum. This study was conducted in strict accordance with the recommendations of the Guide for the Care and Use of Laboratory Animals of the National Institutes of Health, and its protocol was approved by the Animal Research Ethics Board of the Lishui University (Lishui, Zhejiang Province, China. Permit Number: 0803-2019).

### Cell Culture and Induction of DDP-Resistance in OVCAR-3 Cell Line (OVCAR-3/DDP)

To induce the establishment of OVCAR-3/DDP, cells were maintained in DDP that were cultured in Dulbecco’s modified Eagle Medium (DMEM) that contained 10% bovine serum supplemented with 1 × 10^5^ IU/L each of penicillin and streptomycin at 37°C in 5% CO_2_ atmosphere. The OVCAR-3/DDP cells were induced by medium dose of DDP and short-time treatment (19, 20). Briefly, drug concentration was increased by approximately twofold in the initial steps. OVCAR-3 cells in logarithmic growth phase were treated with DDP 5μM for 4 h, then the cells were washed with DDP free medium and continued culture. When the cells returned to the growth stage, the DDP concentration was increased, and the process was repeated. Liquid change and passage were repeated for 36 times, which lasts for 6 months, and these sublines were exposed to 640μM DDP in the end. The IC50 of OVCAR-3/DDP was 502.1μM, compared with parent cell, which was 20.93μM. OVCAR-3/DDP continued to grow in DDP free medium for 2 months and still maintained their drug resistance. Additionally, vehicle-treated parental cell line was kept in culture during this period as a normal cell line.

### Isolation of Ovarian Cancer Stem-Like Cells (CSCs) and Analysis of Cell Surface Marker Expression by Flow Cytometry

As reported in previous studies, CSCs were isolated through magnetic bead sorting by using a magnetic cell sorting system (MACS) (11, 18). Briefly, the cells were harvested from each of the OVCAR-3 cell lines and OVCAR-3/DDP. According to the manufacturer’s instructions, the cells were labeled with CD133 antibodies conjugated to magnetic beads. Subsequently, antibody positive and negative cells were separated using MACS–LS separation columns (Miltenyi Biotec). CD133+ cells were maintained in ultralow attachment plates (Corning Costar Corporation, USA) in knockout DMEM/F12 medium supplemented with 20% knockout serum replacement (Life Technologies), 10 ng/mL basic fibroblast growth factor with 10 ng/mL leukemia inhibitory factor, and 20 ng/mL epidermal growth factor.

To detect the purity and cell surface marker expression of CSCs (CD133+) of OVCAR-3/DDP or OVCAR-3, briefly (11, 18), the CD44 (antibodies, BD Biosciences), CD24 (antibodies, BD Biosciences), CD117 antibodies (Boster Biological Technology, China), and CD133 antibodies (Miltenyi Biotec) were tested by flow cytometry (BD Biosciences, San Jose, CA) according to the manufacturer’s instructions. Cells from each group were collected and centrifuged at 1,500 g for 5 min and suspended with PBS twice. Cells were incubated with antibodies on ice for 40 min in the dark. After washing with cold PBS, the cells were resuspended in 200 μL PBS and subjected to analyses on a flow cytometer. CD24-FITC and CD117-FITC were detected by the FL1A channel, whereas CD44-PE and CD133-PE were detected by FL2A channel. The data were analyzed using the BD FACSDiva™ software to calculate the percentage of double positive.

### Drug Inhibition Rate and IC_50_ Values

CSCs of OVCAR-3 and OVCAR-3/DDP (CSCs/DDP) were treated with 0, 10, 20, 40, and 80 µM of lumiflavin and 0, 40, 80, 160, 320 and 640 µM of DDP for 72 h. Surviving CSCs/DDP were compared with the CSCs combining cell viability/proliferation assays using the CCK-8 assay kit (Dojindo Laboratories, Japan) according to the manufacturer’s instruction. The sensitivity of these paired cell lines is usually determined by exposing them to a range of drug concentrations and then assessing cell viability. The IC_50_ (drug concentration causing 50% growth inhibition) for these paired cell lines can be used to determine the increase in resistance known as the resistance index by the following equation (21):


Resistance index=IC50 of Resistant Cell / IC50 of Normal Cell Line

### Mouse Xenograft Model

All animal studies adhered to the protocols approved by the Animal Research Ethics Board of the Lishui University (Lishui, Zhejiang Province, China. Permit Number: 0803-2019). The CSCs/DDP (1 × 10^5^ cells) were resuspended in 50 μl Matrigel solution (1:1 dilution with DMEM) and injected subcutaneously under the outer skin of 6- to 8-week-old nude female mice (11). When the tumors reached a palpable size (100 mm^3^), the mice were randomly divided into four groups, six mice/each group. Mice were intraperitoneal injected with DDP (CSCs/DDP group, DDP 5 mg/kg, once a week), CSCs/DDP + lumiflavin (4 mg/kg) (DDP 5 mg/kg, once a week; lumiflavin 4 mg/kg, once a day), CSCs/DDP + lumiflavin (8 mg/kg) (DDP 5 mg/kg, once a week; lumiflavin 8 mg/kg, once a day) and CSCs/DDP + lumiflavin (16 mg/kg) (DDP 5 mg/kg, once a week; lumiflavin 16 mg/kg, once a day). All groups of mice were treated for 25 days. The measurement of the length (mm), width (mm) and height (mm) of the tumor masses were performed twice weekly using electronic vernier calipers, and the tumor volumes (mm^3^) were calculated using the following formula: *V* = *L*×*W* × *D* × π/6, where *V* is the volume, *L* is the length, *W* is the width, and *D* is the depth.

### Flow Cytometry Analysis for Cell Apoptosis

CSCs were treated with 0, 80 μM lumiflavin, and CSCs/DDP were treated with 80 μM DDP and 0, 20, 40, and 80 μM lumiflavin for 72h. For FCM analysis, 1×10^6^ cells were harvested, collected by centrifugation (10 min, 2000×g) and washed three times with phosphate-buffered saline (PBS) at 4°C. Prior to analysis, cells were resuspended in binding buffer (10 mM Hepes/NaOH, pH 7.4, 140 mM NaCl, 2.5 mM CaCl). Then, Cells were incubated with 5 μl Annexin V-FITC for 3 min and with 20 ng/mL propidium iodide (Multi Sciences Biotech Co., Ltd., Zhejiang, China) in the dark for 5 min. The apoptosis rate was detected by flow cytometry (BD Biosciences, San Jose, CA). The experiments were repeated three times independently and the results were presented as the mean ± standard deviation.

### Colony Formation Assay

The colony formation capabilities of CSCs and CSCs/DDP were investigated. Briefly, 200 single-cell suspension were resuspended in culture medium into a six-hole plate. CSCs treated with 0 and 80 μM lumiflavin and CSCs/DDP treated with 80 μM DDP and 0, 20, 40, and 80 μM lumiflavin for 14 -15 days until colonies were formed. Colony cells with more than 50 cells were then counted as one positive colony according to laboratory reports (9, 11).

### Cell Mitochondrial Membrane Potential Detected by Flow Cytometry

CSCs were treated with 0 and 80 μM lumiflavin, and CSCs/DDP treated with 80 μM DDP and 0, 20, 40, and 80 μM lumiflavin for 72 h in a 6-well plate. For mitochondrial membrane potential analysis, cells were collected, centrifuged and suspended, and the mitochondrial membrane potential (JC-1) probe (Beyotime Institute of Biotechnology, Jiangsu, China) was mixed. The mitochondrial membrane potential was detected by flow cytometry, according to the JC-1 probe instruction method. The Accuri C6 flow cytometry and the flow cytometry equipped with CellQuest software were used for data analysis.

### RNA Sequencing Analysis

RNA-Seq by Switching Mechanism at 5’ end of RNA Template (SMART) technology was used to study the transcriptome. After CSCs, CSCs/DDP were treated with 80 μm DDP, and CSCs/DDP were treated with 80 μm DDP and 80 μM lumiflavin for 72 h. Next, the cells were collected in TRIzol reagent (Shanghai Jierui Biotech Co., Ltd, Shanghai, China) and then lysed in reaction buffer. Single-cell SMART cDNA was generated by following the SMART-seq protocol (Hangzhou Lianchuan Biotechnology Co., Ltd). The raw data and cluster analysis was performed, and heatmap was generated using the tools in the Lianchuan BioCloud Platform (https://www.lc-bio.cn/overview). The gene ontology (GO) analysis was performed using GOseq packages, and KEGG Orthology (KO) analysis was performed on https://david.ncifcrf.gov/home.jsp (22, 23).

### Western Blot Analysis

Western immunoblotting analysis was carried out for CSCs, CSCs/DDP treated with 80 μM DDP and CSCs/DDP treatment with 80 μM DDP and lumiflavin 80 μM for 72 h, as described previously (24, 25). Cellular proteins were extracted with RIPA buffer [150 mM NaCl, 50 mM Tris (pH 8.0), 10% glycerol, 2% Triton X-100, 1 mM ethylene diamine tetra acetic acid disodium, and a protein inhibitor mixture (Beyotime Biotechnology)]. For immunoblotting, solubilized proteins were loaded on a gel and resolved by 8% – 15% SDS-PAGE. The proteins were then transferred to PVDF membranes and incubated at 4°C overnight with primary antibodies Jagged1 (Jag1,Abcam,1:1000), Jagged2 (Jag2, Abcam,1:1000), Delta-like canonical notch ligand 1 (Dll1, Abcam,1:500), Delta-like canonical notch ligand 3 (Dll3, Cell Signaling Technology,1:1000), Delta-like canonical notch ligand 4 (Dll4, Cell Signaling Technology,1:1000), Notch1 (Abcam,1:1000), Notch2 (Abcam,1:5000), Notch3(Santa Cruz, 1:500), Notch4 (Santa Cruz, 1:500) or NICD (Cell Signaling Technology, 1:1000). Membranes were washed and incubated with anti-rabbit/anti-mouse antibodies (MultiSciences Biotech, 1:5000) for 1 h at room temperature. Chemiluminescent images of the blots were finally captured using a ChemiDoc System (Bio-Rad, USA). ImageJ software was used to calculate relative protein expression.

### Data Analysis

All experiments were repeated at least three times and the data are presented as the mean ± S.D. Differences among data groups were evaluated for significance using the Student’s *t* test of unpaired data (two‐tailed). For animal studies, the data are presented as the mean ± S.E.M. The *F* test for the homogeneity of variance was conducted. The tumor volume was analyzed with one‐way ANOVA using the software SPSS 11.5 for Windows (Chicago, IL, USA). Significant and highly significant differences were considered at *P* < 0.05 and *P* < 0.01, respectively.

## Results

### Lumiflavin Treatment Reduces the Formation of Drug Resistance and Proportion of CSCs in OVCAR-3

**Figure 2:**
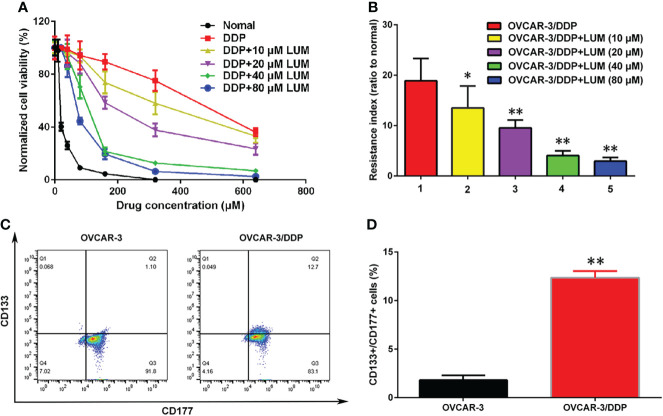
Lumiflavin treatment reduces the development of DDP resistance of ovarian cancer OVCAR-3 cells line that is associated with cancer stem cells (CSCs). DDP resistance of ovarian cancer OVCAR-3 cell lines were induced by gradient concentration increment method (26), and treated with 10, 20, 40, and 80 μm lumiflavin intervention simultaneously. The cell inhibition rate and resistance index of OVCAR-3cells to DDP were detected by CCK-8 assay kit. The proportion of CSCs (CD133+/CD177+ double positive cells) in OVCAR-3 cells was detected by flow cytometry. **(A)** Inhibitory curves of lumiflavin and DDP on OVCAR-3 and OVCAR-3/DDP cells. **(B)** Resistance index of OVCAR-3/DDP cells compared with OVCAR-3 cells. **(C)** Flow detection results of CD133+/CD117+ cells ratio of OVCAR-3 and OVCAR-3/DDP cells. **(D)** Statistical analysis graph of **(C)**. Mean ± SD (*n* = 3). **P < 0.01 difference between groups; *P < 0.05 difference between groups. DDP, cisplatin; OVCAR-3/DDP, DDP resistant OVCAR-3 cell lines; LUM, lumiflavin.

In this study, we further investigated the effect of lumiflavin on the development of resistance in ovarian cancer cells. The drug resistance of ovarian cancer cell line OVCAR 3 (OVCAR 3/DDP) was induced by gradient concentration increase method (26), while treating with 0, 10, 20, 40, and 80 μm lumiflavin intervention. At the end, OVCAR-3 and OVCAR-3/DDP intervened with different concentrations of lumiflavin were treated with 0, 40, 80, 160, 320 and 640 μM DDP for 72 h, respectively. Cell viability was detected with CCK-8 assay kit. The results showed that the cell viability rate of the OVCAR-3 cells decreased in a dose-dependent manner with the increase in DDP concentration. Compared with normal OVCAR-3, the cell viability rate of the OVCAR-3 cells at 20 μM DDP and 160 μM DDP was 44% and 7%, respectively. In contrast, the cell viability rate of OVCAR-3/DDP at 160 μM DDP and 640 μM DDP was 85% and 38%, respectively, thereby indicating the significant resistance to DDP. The treatment of lumiflavin could reduce the drug resistance of OVCAR-3/DDP cells significantly, showing that after treatment with lumiflavin at 10, 20, 40, and 80 μM, the cell survival rate was 66%, 60%, 26% and 17% under 160 μM DDP, and 27%, 24%, 11% and 2% under 640 μM DDP, respectively, compared with OVCAR-3/DDP. The resistance index of the OVCAR-3/DDP cells after 0, 10, 20, 40, and 80 μM lumiflavin intervention groups was 18.9, 13.5, 9.5, 4.1, and 3.0, respectively, compared with OVCAR-3/DDP (**Figures 2A, B**).

The proportion of CSCs (CD133+CD177+ double positive cells) in OVCAR-3 and OVCAR-3/DDP was determined by flow cytometry. The results showed that compared with OVCAR-3 cells, the proportion of CSCs/DDP in OVCAR-3/DDP cells increased (*P*<0.01) (**Figures 2C, D**).

### Lumiflavin Enhances the Chemotherapy Effect of DDP on CSCs/DDP

**Figure 3:**
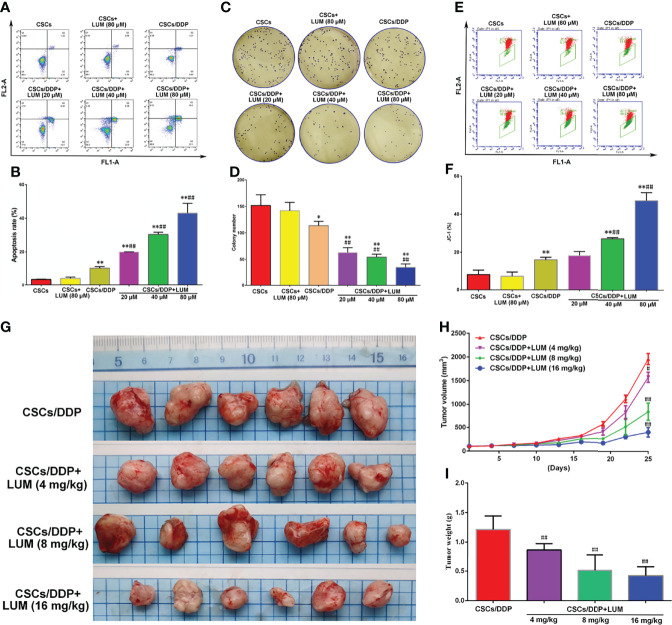
Lumiflavin enhances the chemotherapy effect of DDP on DDP-resistant cancer stem cells (CSCs/DDP). CSCs, CSCs/DDP were treated with 80 μM DDP, and CSCs/DDP were treated with 80 μM DDP and 20, 40, 80 μM lumiflavin for 72 h. Apoptosis and clone formation ability were detected. Moreover cell mitochondrial membrane potential were tested by JC-1 prob (27, 28). CSCs/DDP were subcutaneously inoculated in nude mice and treated with DDP (DDP 5 mg/kg, once a week) combined with lumiflavin for 25 d. Tumor weight and inhibited tumor growth curve were measured. **(A)** Flow cytometry of cell apoptosis as detected using Annexin V-FITC. **(B)** Statistical analysis graph of **(A, C)** Images of colony formation assay. **(D)** Statistical analysis graph of **(C, E)** Flow cytometry scatter plot of the potential of the mitochondrial membrane (Δψm) of cells as detected through flow cytometry by using a JC‐1 probe. **(F)** Statistical analysis graph of **(E)**. **(G)** Photograph of tumors. **(H)** Tumor growth curve of nude mice. **(I)** Statistical graph of tumor weight. Mean ± SD (*n* = 3, cells; *n* = 6, mice). *P < 0.05 compared with the CSC group; **P < 0.01 compared with the CSC group; ^##^*P* < 0.01 compared with CSC/DDP group. CSCs, CSCs from OVCAR-3 cell lines; CSCs/DDP, CSCs from DDP resistant OVCAR-3 cell lines; LUM, lumiflavin.

CSCs/DDP were separated from OVCAR-3/DDP to further study whether the effect of lumiflavin on OVCAR-3/DDP cells is associated with CSCs. CSCs, CSCs/DDP were treated with 80 μM DDP, and CSCs/DDP were treated with 80 μM DDP and 20, 40, 80 μM lumiflavin for 72 h. The apoptosis and clone formation ability were detected. The flow cytometry results of flow cytometry showed that the apoptosis rate of the OVCAR-3/DDP cells was 3.6% after 80 μM DDP treatment for 72 h, while whereas combined with 20, 40, and 80 μM lumiflavin, the apoptosis rate was 20%, 30.4%, and 43%, respectively (compared with the OVCAR-3/DDP cells, *P* < 0.01). The results of clone formation experiment showed that after 80 μM DDP intervention, the number of clones was 113.7/well in OVCAR-3/DDP cells, and 63.0/well, 54.3/well and 35.0/well after the combined treatment with 20, 40, and 80 μM lumiflavin, respectively (compared with the OVCAR-3/DDP cells, *P* < 0.01). These results showed that lumiflavin increased CSCs/DDP apoptosis rate and reduced cell clone formation ability in a dose-dependent manner (**Figures 3A–D**).

JC-1 is a cationic dye used for the detection of mitochondrial membrane potential, which can enter the negatively charged mitochondria and accumulate to form aggregates (27). Excited by fluorescence at 488 nm wavelength, JC-1 emits a red spectrum. The decrease in mitochondrial membrane potential will lead to the dissociation of JC-1 into monomer, and the green light will be emitted. Flow cytometry shows that the FL1-A signal is enhanced and the FL2-A signal is significantly decreased (28). The flow cytometry results showed that the proportion of JC-1 in OVCAR-3/DDP cells was 16.1% after 80 μM DDP treatment for 72 h, whereas combined with 20, 40, and 80 μM lumiflavin, proportions of JC-1 were 18.4%, 27.0%, and 47.1%, respectively (compared with the OVCAR-3/DDP cells, *P* < 0.01). This study showed that lumiflavin treatment increased the proportion of JC-1 in CSCs/DDP, thereby suggesting that mitochondrial membrane potential (*Δψm*) is decreased and induced CSC apoptosis (**Figures 3E, F**).

To further explore the effect of lumiflavin on CSCs/DDP *in vivo*, CSCs/DDP were subcutaneously inoculated in nude mice and treated with DDP (5 mg/kg) combined with lumiflavin (4, 8 and 16 mg/kg) for 25 d. The results showed that lumiflavin combined with DDP reduced tumor weight and inhibited tumor growth curve in a dose-dependent manner, compared with DDP alone (**Figures 3G–I**).

### Lumiflavin Treatment Affects the Phenotypic Differentiation of CSCs/DDP

**Figure 4:**
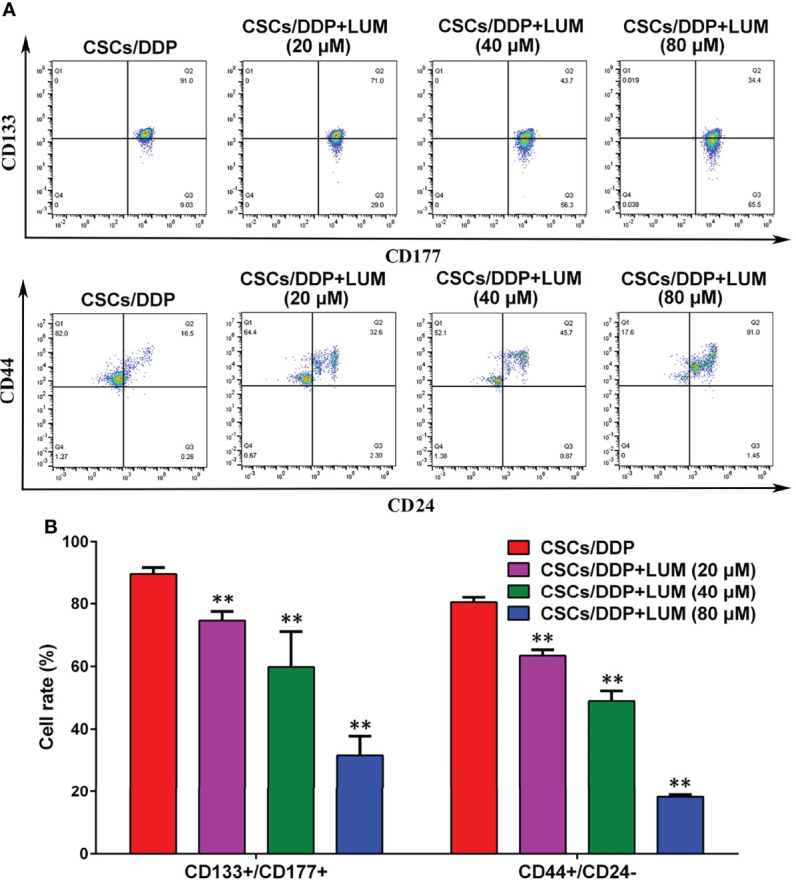
Effects of lumiflavin treatment on phenotypic differentiation of DDP-resistant cancer stem cells (CSCs/DDP). CSCs, CSCs/DDP were treated with 80 μM DDP, and CSCs/DDP were treated with 80 μM DDP and 20, 40, 80 μM lumiflavin for 72 h. Co-expression of CD133+/CD117+, CD44+/CD177, and CD44+/CD24– of CSCs/DDP were detected through flow cytometry. **(A)** Flow detection results of CD133+/CD117+, CD44+/CD177, and CD44+/CD24- cells of CSCs/DDP. **(B)** Statistical analysis graph of **(A)**. **P < 0.01 between groups. Mean ± SD (*n* = 3). CSCs/DDP, CSCs from DDP resistant OVCAR-3 cell lines; LUM, lumiflavin.

CD133 and CD177 are highly expressed in CSCs and are required for the maintenance of CSCs (29). As illustrated in **Figure 4**, after 80 μM DDP treatment, the proportion of CD133+/CD177+ cells in CSCs/DDP cells was 89.7%, whereas after combined treatment with 20, 40, and 80 μM lumiflavin, the proportion became 74.6%, 59.8%, and 31.6%, respectively. In addition, we also investigated the proportion of CD44+/CD24- cells, an important characteristic of CSCs (30), after 20, 40, and 80 μM lumiflavin treatment, the proportion was 63.4%, 49.0% and 18.2%, respectively, compared with 80.5% for CSCs/DDP cells. The results showed that after 72 h treatment with lumiflavin, the proportions of both types of cells decreased and showed dose dependence (compared with CSCs/DDP group, *P* < 0.01). These results suggested that lumiflavin combined with DDP could induce cell differentiation and inhibit the phenotypic maintenance ability of CSCs/DDP, as shown in **Figures 4A, B**.

### Bioinformatics Analysis of the RNA-Seq Assay

**Figure 5:**
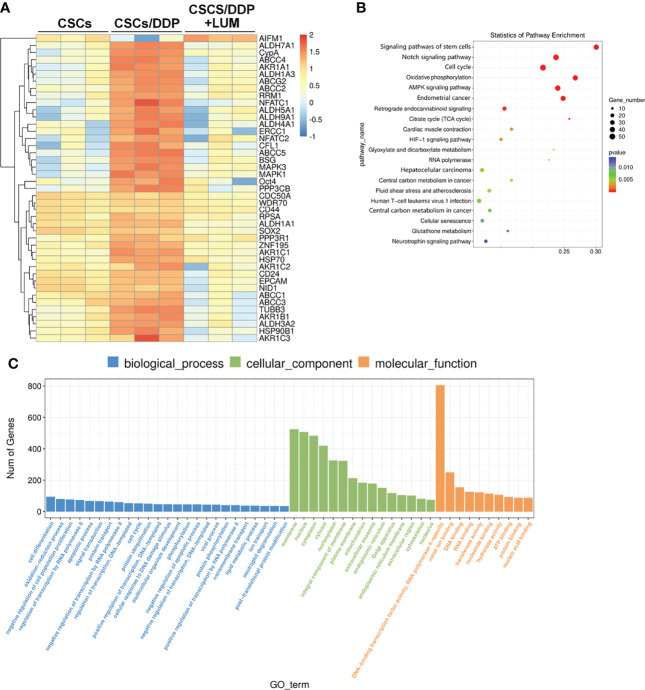
RNA-seq analysis of transcriptome changes in lumiflavin on DDP-resistant cancer stem cells (CSCs/DDP). CSCs, CSCs/DDP were treated with 80 μM DDP, and CSCs/DDP were treated with 80 μM DDP and 80 μM lumiflavin for 72 h. The transcriptome changes were detected by RNA-Seq assay. **(A)** Enrichment analysis of RNA-Seq assay. **(B)** Scatter diagram of KEGG enrichment analysis results of RNA-Seq assay. **(C)** Gene ontology analysis was utilized to evaluate the canonical pathways in the RNA-Seq assay results. Mean ± SD (n = 3). CSCs, CSCs were separated from OVCAR-3 cells line; CSCs/DDP, CSCs were separated from OVCAR-3/DDP cells line; LUM, lumiflavin.

To further explore the molecular mechanism of the combined effect of lumiflavin and DDP on CSCs/DDP, RNA-Seq was used to detect the transcriptome changes of CSCs/DDP treated with 80 μM lumiflavin and 80 μM DDP for 72 h. An example heat map showing the Log10 FC expression values of differentially expressed mRNA were plotted by heat map. We analyzed the statistical enrichment of differentially expressed genes in KEGG pathway. Based on the results of significantly differentially expressed analysis, six pathways were significantly enriched (*P* < 0.05). The most enriched pathways included stem cell signaling pathway, Notch signaling pathway, cell cycle, oxidative phosphorylation, and AMPK signaling pathway. Next, we performed GO analysis on all the differential mRNA, and the results showed that the differential gene functions focused on cell differentiation, oxidation–reduction process, population proliferation, as well as DNA-binding transcription factor activity, RNA polymerase II-specific. Among the cell components, it affects the nucleus, membrane, and cytoplasm (**Figures 5A–C**).

### Lumiflavin Downregulated the Protein Expression of Notch Signaling Pathway in CSCs/DDP

**Figure 6:**
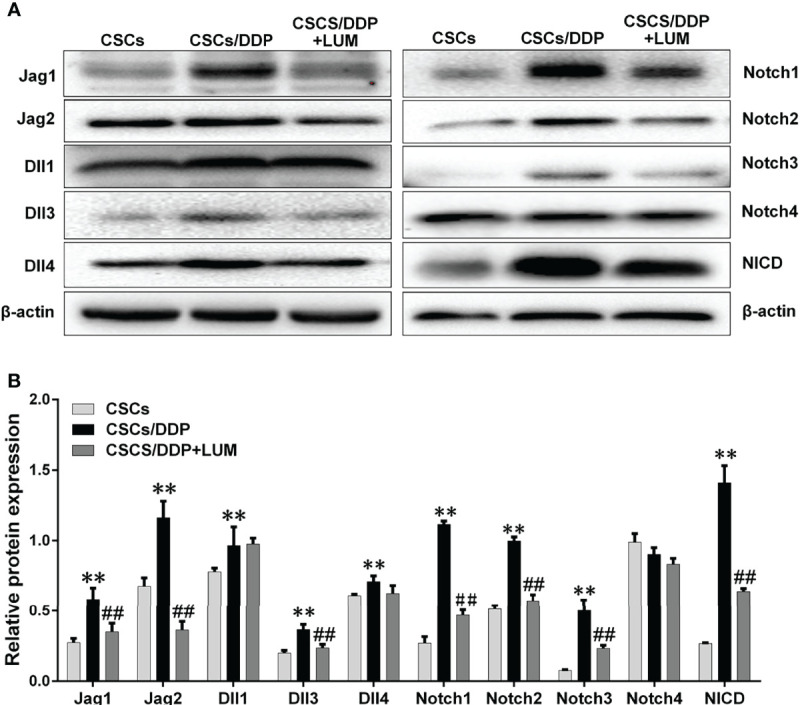
Lumiflavin downregulated the protein expression of Notch signaling pathway in DDP-resistant cancer stem cells (CSCs/DDP). CSCs, CSCs/DDP were treated with 80 μM DDP, and CSCs/DDP were treated with 80 μM DDP and 80 μM lumiflavin for 72 h. The expression levels of Jag1, Jag2, Dll1, Dll3, Dll4, Notch1, Notch2, Notch3, Notch4, NICD, and β-actin were analyzed *via* Western blot by using specific antibodies. **(A)** Electrophoretograms of proteins. **(B)** Graph of relative protein expression as determined by Western blot analysis. **P < 0.01 compared with CSC group; ^##^*P* < 0.01 compared with CSCs/DDP group. Mean ± SD (*n* = 5). CSCs, CSCs were separated from OVCAR-3 cells line; CSCs/DDP, CSCs were separated from OVCAR-3/DDP cells line; LUM, lumiflavin.

Previous RNA-Seq assay revealed that the effect of lumiflavin on CSCs is related to the Notch signaling pathway, stem cell signaling pathway, cell cycle, oxidative phosphorylation, and AMPK signaling pathway. Moreover, Notch has important interactions with stem cell and AMPK signaling pathway and stem cell signaling pathway (31, 32). Next, CSCs, CSCs/DDP were treated with 80 μM DDP, and CSCs/DDP were treated with 80 μM DDP and 80 μM lumiflavin for 72 h. Western blotting was used to study the effects of lumiflavin on Notch signaling related proteins in ovarian cancer cell line CSCs/DDP. Moreover, the results show that Notch-1, Notch-2, Notch-3, and Notch ligands, just as Jag1, 2 and Dll 1, 3, 4 were increased in CSCs/DDP, compared with CSCs, whereas the expressions of Notch-1, Notch-2, Notch-3 and Jag1, 2 and Dll 3 were downregulated following lumiflavin treatment. Further confirmation showed that NICD expression was increased in CSCs/DDP, compared with CSCs, and reduced expression in CSCs/DDP treatment with lumiflavin (**Figures 6A, B**).

## Discussion

Cell line models have proven to be an effective tool for ovarian cancer research, and the study of the mechanism of drug-resistant cell lines will contribute to the development of anti-cancer drugs. Furthermore, cell lines taken from cancer patients before and after chemotherapy, are ideal models for drug resistance development (33, 34). In this study, drug-resistant ovarian cancer cell lines, such as OVCAR-3/DDP were established through induction, and the drug resistance index showed that the IC50 concentration of OVCAR-3/DDP was more than 18 times that of OVCAR-3cells. Simultaneous intervention with four concentrations of lumiflavin was found to dose-dependently shift the resistance curve of OVCAR-3/DDP and reduce the resistance index. The results of experiments *in vitro* have shown that the development of drug resistance can be weakened by simultaneous treatment with lumiflavin.

The hypothesized properties of CSCs subpopulations have been used to help explain the phenomena observed in cancer biology, and various approaches to CSCs in the search for these “cancers” have been applied to hematologic diseases and solid malignancies (35, 36). CSCs can be studied in two main methods. One is to isolate cells, such as CD133+, CD177+, or CD44+/CD24-, that have stem-like characteristics (37–40). Another approach is to take advantage of the CSCs’ ability to excrete fluorescent dyes and obtain so-called “Side population (SP) cell” as study cells by flow cytometry sorting, and recent reports suggested that SP cells may represent different types of CSCs malignancies (41, 42). In this study, the proportion of CSCs (CD133+/CD177+) in OVCAR-3/DDP was significantly increased (13.1%) compared with that of OVCAR-3 (1.75%), which was similar to that in related studies (43, 44). These results suggest that CSCs acquire drug-resistance character more easily than normal non-cancer stem cells; and in the process of DDP chemotherapy, non-cancer stem cells are easier to be killed than CSCs. CSCs play a significant role in the induction of drug-resistant cells.

Apoptosis rate is an important indicator to reflect the killing effect of chemotherapy drugs, and the colony-forming capacity is an important characteristic of CSCs (45).In this study, lumiflavin increased the apoptosis rate and reduced the colony-forming capacity of CSCs/DDP. These results suggested that the effect of lumiflavin on OVCAR-3/DDP was related to CSCs/DDP.

The ability of CSCs to resist chemotherapy damage is stronger than that of ordinary cancer cells, and previous studies have found that it is related to the ability of CSCs to resist oxidative damage. Moreover, lumiflavin can attenuate the antioxidant damage ability of CSCs (11). This study confirmed that lumiflavin has the same role in oxidative damage in CSCs/DDP. Decreased mitochondrial capacity to resist oxidative damage leads to the accumulation of ROS, which is a key event in inducing apoptosis (46). The change in mitochondrial permeability and the decrease in transmembrane potential after mitochondrial damage are the initial events of the cascade of apoptosis, and mitochondrial dissipation leads to irreversible apoptosis (47, 48). In this study, lumiflavin-assisted DDP reduced mitochondrial transmembrane potential of CSCs/DDP, suggesting that the synergic effect of lumiflavin and DDP was related to increased mitochondrial damage. This point is consistent with our previous researches (11, 18).

The anti-injury characteristics of CSCs make it difficult for ordinary chemotherapy to kill CSCs effectively, so the differentiation of CSCs into normal cancer cells is a good strategy to kill CSCs effectively (12, 49). The flow cytometry results showed that the proportion of CD133+/CD177+ and CD44+/CD24- in CSCs/DDP decreased after lumiflavin intervention, suggesting that the synergistic effect of lumiflavin and DDP may be related to its differentiation. Thus, transcriptome sequencing analysis was performed on CSCs/DDP treated with lumiflavin, we found that the differences in mRNA expression were mainly concentrated in the Notch signaling pathway, which is the key pathway of CSCs characteristics (50, 51). Moreover, the biological function analysis of gene differences revealed that these genes concentrated in cell cycle and oxidative phosphorylation pathway. Together, they show that the effects of lumiflavin on CSCs/DDP are concentrated on the cell differentiation.

RNA-seq analysis showed significant changes in Notch signaling pathway, stem cell signaling pathway, cell cycle, oxidative phosphorylation and AMPK signaling pathway. Previous studies have confirmed that the effect of lumiflavin is related to oxidative phosphorylation and oxidative stress (11). Moreover, Notch is closely related to stem cell signaling pathway, AMPK signaling pathway and cell cycle (31, 52, 53). Notch pathway is also critical for CSC differentiation and drug resistance in cancer (54, 55). Therefore, we speculate the key role of the Notch pathway and further investigate its changes. As shown in **Figure 5**, compared with normal CSCs, the expression of ligands (e.g., Jag1, Jag2, Dll1, Dll3 and Dll14) of four Notch receptors (e.g., Notches 1 to 4) and NICD were up regulated in CSCs/DDP, compared with CSCs. It is suggested that Notch signaling pathway is enhanced and related to drug resistance of CSCs. However, these protein expressions were decreased, except DII 4 and Notch4, in lumiflavin treatment group, suggesting that the role of lumiflavin may be related to Notch pathway in CSCs/DDP phenotypic transformation.

In this study, the effect of lumiflavin on the development of drug resistance in CSCs of OVCAR-3 cell was studied, suggesting that lumiflavin can slow down the progress of CSCs/DDP and increase the killing effect of DDP on CSCs/DDP. It was preliminarily shown to be related to the differentiation of CSCs/DDP, but this study still had the following deficiencies: 1) Sequencing results showed that other important signaling pathways, such as oxidative phosphorylation and AMPK signaling pathway, were significantly altered, and the roles of these pathways in this study have not been explored yet; and 2) CSCs will be subject to many influencing factors *in vivo*, which will affect the differentiation and drug resistance of CSCs. These problems will be elaborated in the future research.

## Data Availability Statement

The original contributions presented in the study are publicly available. This data can be found here: https://www.ncbi.nlm.nih.gov/bioproject/PRJNA810781.

## Ethics Statement

The animal study was reviewed and approved by the Animal Research Ethics Board of the Lishui University.

## Author Contributions

RY conducted animal experiments and most of cell experiments and drafted the manuscript. BL conducted immunoassay and cell assay, participated in the animal experiments. FX participated in the design of the study and performed the statistical analysis. MY and SW participated in the writing of the manuscript. SW participated in the design of the study and proofread the manuscript. SZ participated in its design and the sequence alignment and drafted the manuscript. All authors read and approved the final manuscript.

## Funding

This work was supported by grants from the Fund of Natural Science Foundation of Zhejiang Province, China [NO: LY20H310007], Department of Education of Zhejiang Province [NO: Y201839774], and Key project of Taizhou Vocational and Technical College [NO: 2022ZD02].

## Conflict of Interest

The authors declare that the research was conducted in the absence of any commercial or financial relationships that could be construed as a potential conflict of interest.

## Publisher’s Note

All claims expressed in this article are solely those of the authors and do not necessarily represent those of their affiliated organizations, or those of the publisher, the editors and the reviewers. Any product that may be evaluated in this article, or claim that may be made by its manufacturer, is not guaranteed or endorsed by the publisher.
